# Allelic variants of *IL1R1 *gene associate with severe hand osteoarthritis

**DOI:** 10.1186/1471-2350-11-50

**Published:** 2010-03-30

**Authors:** Annu Näkki, Sanna T Kouhia, Janna Saarela, Arsi Harilainen, Kaj Tallroth, Tapio Videman, Michele C Battié, Jaakko Kaprio, Leena Peltonen, Urho M Kujala

**Affiliations:** 1 Institute for Molecular Medicine Finland FIMM, University of Helsinki, Helsinki, Finland; 2 Public Health Genomics Unit, National Institute for Health and Welfare, Helsinki, Finland; 3 Department of Public Health, University of Helsinki, Helsinki, Finland; 4 ORTON Orthopedic Hospital, Invalid Foundation, Helsinki, Finland; 5 Faculty of Rehabilitation Medicine, University of Alberta, Edmonton, Canada; 6 Department of Mental Health, National Institute for Health and Welfare, Helsinki, Finland; 7 Wellcome Trust Sanger Institute, Cambridge, United Kingdom; 8 The Broad Institute of MIT and Harvard, Boston, MA, USA; 9 Department of Health Sciences, University of Jyväskylä, Jyväskylä, Finland; 10 Department of Medical Genetics, University of Helsinki, Helsinki, Finland

## Abstract

**Background:**

In search for genes predisposing to osteoarthritis (OA), several genome wide scans have provided evidence for linkage on 2q. In this study we targeted a 470 kb region on 2q11.2 presenting the locus with most evidence for linkage to severe OA of distal interphalangeal joints (DIP) in our genome wide scan families.

**Methods:**

We genotyped 32 single nucleotide polymorphisms (SNPs) in this 470 kb region comprising six genes belonging to the interleukin 1 superfamily and monitored for association with individual SNPs and SNP haplotypes among severe familial hand OA cases (material extended from our previous linkage study; n = 134), unrelated end-stage bilateral primary knee OA cases (n = 113), and population based controls (n = 436).

**Results:**

Four SNPs in the *IL1R1 *gene, mapping to a 125 kb LD block, provided evidence for association with hand OA in family-based and case-control analysis, the strongest association being with SNP rs2287047 (p-value = 0.0009).

**Conclusions:**

This study demonstrates an association between severe hand OA and *IL1R1 *gene. This gene represents a highly relevant biological candidate since it encodes protein that is a known modulator of inflammatory processes associated with joint destruction and resides within a locus providing consistent evidence for linkage to hand OA. As the observed association did not fully explain the linkage obtained in the previous study, it is plausible that also other variants in this genome region predispose to hand OA.

## Background

Osteoarthritis (OA) is a complex late onset disease characterized by the destruction of joints and it can occur in one or multiple joint groups as hand and knee OA often do [1-3]. The most frequent clinical findings in OA are pain associated with loading and limited range of motion in the joints at later stages. Typical radiographic findings, narrowing of the intra-articular space and osteophytes, are considered superior to clinical manifestations in the diagnostic definition [4]. There is a strong genetic effect on hand and knee OA, with heritability ranging between 39%-65% independent of known environmental or demographic confounders [5]. Based on a Finnish study [2], the genetic effects seem to be more prominent in females.

Our genome wide linkage analysis in OA families identified chromosome 2q11-q21 locus as the major locus for severe hand OA in Finnish families [6]. The same locus has been identified in screens of rare multiplex pedigrees and sibpairs from more heterogeneous populations [7,8], and in a large population-based twin cohort [9]. The 2q11-q21 locus harbours the interleukin 1 (IL-1) gene cluster. The marker providing strongest evidence for linkage in Finnish distal interphalangeal joint (DIP) OA families resides within the *IL1R1 *gene (LOD score 2.34), and some evidence for a shared haplotype among the affected individuals was observed with markers D2S2264, IL1R1, D2S373, and D2S1789 (p-value of 0.012) [6]. Further, a handful of association studies have shown a potential role for interleukin-1 gene family in knee and hip OA [10-12].

In the human genome, *IL1R2*, *IL1R1*, *IL1RL2*, *IL1RL1*, *IL18R1 *and *IL18RAP *are located in a 470 kb cluster on 2q11. Genes coding for most other IL-1 ligands are also clustered on chromosome 2 [13]. IL-1 is a pleiotropic cytokine involved in the initiation of inflammatory and immune responses. Using gene names also for their protein products, a complex of IL-1 (IL1A or IL1B), IL1RI and IL1RAP are needed for signal transduction. There are other interleukin ligand-receptor complexes leading to NF-κB activation too [14-16]. Therapeutic effect of IL-1 receptor antagonist (IL1RN) gene transfer has been confirmed in different experimental models of OA [17].

Our aim was to study more closely the 2q11-q21 locus, major locus for severe hand OA in our previous study, using SNPs and SNP haplotypes in extended hand OA families (including siblings with a severe, radiologically defined bilateral DIP OA used in our genome scan [6]), and in a set of severe primary bilateral knee OA patients. We targeted a 470 kb region in chromosome 2q11.2 by genotyping 32 SNP markers in total of 220 DNA samples from individuals affected with OA and in 436 population controls.

## Methods

### Subjects

Our hand OA material was based on the set of radiologically verified severe DIP OA families from our previous study described in more detail in Leppävuori et al. [6] and summarised in Table 1. DNA samples of 86 severe radiologically verified bilateral DIP OA cases were available from our previous study [6]. A radiologist had graded the osteoarthritic changes in the hand x-rays for DIP, PIP, MCP joints (according to [18]). Data for reported height and weight were available. Subjects with rheumatoid arthritis (RA) were excluded. Inclusion criteria were 3rd or 4th degree Kellgren and Lawrence (K/L) radiographic OA in DIP joints (bilateral) and a positive family history of OA, with at least one sibling with DIP OA. The other siblings were included as affected individuals if they had a K/L score ≥ 2 in DIP joints. Additionally, 21 unaffected family members derived from 40 families were available for this study. Study set was further extended by 17 radiologically verified severe DIP OA cases collected from the ORTON Orthopaedic Hospital (Helsinki, Finland) with the same radiographic criteria as used in selecting the index cases in initial material (Table 1) and also 4 affected siblings. The average age at grading of hand X-rays was 62 years (SD 9 y). For more about diagnostics and epidemiology of hand OA, see Haara et al.[19].

**Table 1:** The number of cases and controls according to case definition and sample provenance.

	Number of subjects		
	
	Total	Male	Female
Hand OA material	168^a^	36	132
44 families with ≥ 2 DIP OA cases^b^	116	22	94
4 families with 1 DIP OA case^b^	15	5	10
Additional radiologically verified DIP OA cases^b^	9	4	5
Physician diagnosed hand OA cases^c^	28	5	23
Knee OA material^b^	113	27	86
Controls	436	277	159

Total	689	335	354

^a ^= The total number of affected individuals was 134 of which 85 were unrelated

^b ^= Radiologically verified OA cases

^c ^= Physician diagnosed hand OA; this group is also a part of radiological knee OA material

To collect knee OA material, we reviewed case reports of 852 patients visiting ORTON Orthopaedic Hospital between 1994-2001 having knee OA and identified 220 patients with primary bilateral knee OA severe enough to fulfill the criteria for knee arthroplasty: pain, walking disability and radiologically at least stage 3/4 osteoarthritic changes according to Kellgren and Lawrence classification [20]. Of the 220 patients, we reached 113 subjects, who had not had a major knee trauma and volunteered to participate by providing a DNA sample (Table 1). Knee OA patients' pain or other OA symptoms began at a mean age of 52 y (SD 12 y), and mean age at first arthroplasty was 67 y (SD 8). 28 patients had physician-diagnosed hand OA and were also included in the hand OA material to increase statistical power.

The control subjects (n = 436, aged 35-70) were selected from the Finnish twin cohort [21]. Approximately half of this control material (n = 210) was selected from the Finnish twin cohort [21] with inclusion criteria of born in 1938-1941, responded to a questionnaire in 1996-1997, did not have diagnosis of OA and gave DNA samples for analyses. One twin from each twin pair was included in the control group if neither twin had physician diagnosed OA or RA, and neither twin reported that their mother, father, co-twin or any other sibling had OA or RA. The remaining half (n = 226) of the controls were subjects of Twin Spine study which is part of the Finnish twin cohort study. The Twin Spine study consists of 300 male twin pairs selected on the basis of putative risk factors for low back pain and disc degeneration (not particularly suffering from either) as described elsewhere [22,23]. Subjects with a physician-diagnosed OA were excluded, and only one twin from a pair was included. The study was approved by the ethics committee of the Helsinki metropolitan hospital region and all individuals gave their informed consent.

### Methods and statistical analyses

**Figure 1:**
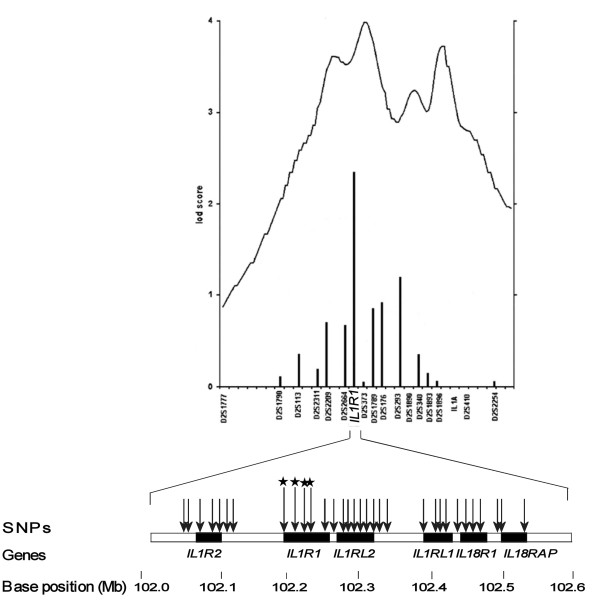
**SNP selection based on the results of genome wide scan by Leppävuori et al**. [6]. Pairwise dominant Zmax values (columns); -log10 P values measuring the maximum number of genes among affecteds IBD to any founder gene (continuous line); selected SNPs markers for this study (arrows); association to hand OA (p-value < 0.05) (star).

A set of 49 SNPs with a minimum heterozygosity of 0.2 in the Caucasian population were selected from SeattleSNP [24] and Snpper CHIP Bioinformatics Tools [25] databases to cover the 470 kb target region with approximately 10-kb SNP spacing (Fig. 1). These SNPs were genotyped using the MassARRAY^® ^system (Sequenom, San Diego, CA, USA) as recommended by the manufacturer with additional quality assessment steps described in more detail in Silander et al. [26]. The genotyping was done in multiplexes of 1-5 SNPs using hME assays in 384-well plates. Each SNP multiplex was validated by genotyping 81 trio samples (i.e. 27 families with two parents and a child) to control for Mendelian incompatibilities using Pedcheck 1.1 program [27]. Each 384-well plate contained 8 duplicate samples and 8 non-template wells for quality assessment. If the genotypes could not be reliably called, if there were unexplainable errors in Mendelian inheritance or between duplicate samples, or genotypes in the non-template wells, SNP was excluded from the study. Further, only SNPs with call rate of over 90% were included. A total of 32 SNP passed the preset quality criteria and were included in the further analysis. All included SNPs showed HW p-values of more than 0.1.

Additionally using a microarray based allele specific primer extension method [28], we genotyped eight SNPs in more studied area in chromosome 2q harbouring the *TRAP1, IL1A, IL1B, IL1F5 *and *IL1RN *genes (rs1030878, rs2071375, rs1800587 (SNP genotyped approximately in half of the controls, n = 169), rs1143634, rs1530551, rs2234677, rs315952, and rs9005) three of which were previously analysed by Smith and colleagues [29]. In this additional analysis we lacked 67 individuals (one hand OA case, four unaffected hand OA family members, 21 knee OA cases and 41 controls).

Deviation from the Hardy-Weinberg equilibrium was tested using Pearson's χ^2 ^test in the control sample. For hand OA families, Pedcheck 1.1 [27] was used to rule out Mendelian errors. The degree of pairwise linkage disequilibrium (LD) was calculated for each pair of SNPs in control samples using the Haploview program [30]. Both r^2 ^and D' measures of LD were studied. Then, evidence for association was evaluated in hand OA and knee OA subgroups using the Pseudomarker 0.9.7 beta program which can utilize the information from family and case-control settings [31]. We monitored for LD with the disease assuming linkage. Results were confirmed by traditional χ^2 ^analysis (including only an index case of each hand OA family) or by Fisher's test when less than five individuals were carrying the same genotype. The Pseudomarker analysis was performed additionally for females only in both disease groups. The number of males was too small in both case groups for separate analysis.

To evaluate the power in negative findings for knee OA group, we calculated the amount of cases required to have 90% power to detect similar association as with the hand OA group and the power with 113 cases using the Genetic Power Calculator [32]. The SNPSpD method [33] with modifications by Li and Ji [34] was used to calculate the p-value threshold for 5% significance [35].

Next using the Haploview program [30], we performed haplotype association test in the area that showed the strongest evidence for association with individual SNPs located in a single LD block measured by r^2^, covering the *IL1R1 *gene (rs1465325, rs956730, rs3917225, rs2287047, rs3771200) (index case per family in hand OA group). We further estimated haplotypes for each subject individually using the Phase2 program [36,37] for calculating odds ratios (OR) for associated haplotypes (index case per family in hand OA group). A minimum probability of ≥ 0.60 was requested for the haplotypes, and alleles with a frequency of over 0.05 in the control sample were each analyzed separately and the less common haplotype alleles were grouped together. Hardy-Weinberg equilibrium was calculated for tested haplotypes to confirm the correctness of the haplotype allele distribution. For further information on the haplotype association in the hand OA group, we conducted haplotype association test in the associated area for SNPs in LD measured by D' solid spine of LD (rs1465325, rs956730, rs3917225, rs2287047, rs3771200, rs2241132, rs870684, rs1922290 and rs1922295) using the Haploview program [30].

Finally, to study the linkage between hand OA and SNPs showing the strongest association with hand OA, the Pseudomarker program was used. Then, to assess if affected individuals with a certain genotype contribute more than expected to original linkage in the region, we used the genotype-IBD Sharing Test (GIST) software package [38,39]. Analysis was performed with the families used in the original genome-wide linkage scan and also included in this study (23 families). We used genotype data for SNPs showing the most significant association and single point NPL scores for the original "IL1R1" microsatellite marker for each family obtained from the Merlin program [40,41].

## Results

**Figure 2:**
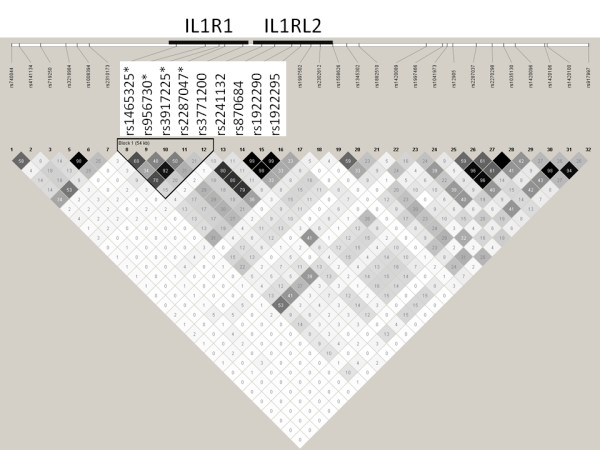
**LD structure and the degree of linkage disequilibrium (r^2^) for each SNP pair in control sample **[30]. SNPs associating to hand OA (stars) and r^2 ^haplotype block.

A set of 32 SNPs mapping within a 470 kb region on 2q11.2 presenting the locus with most evidence for linkage to severe hand OA in our previous genome wide scan families (Fig. 1) was genotyped in severe hand OA cases (n = 134), end-stage bilateral primary knee OA cases (n = 113) and controls (n = 436) (Table 1). According to SNPSpD, the effective number of independent SNP loci was 23.9 thus the experiment-wide significance threshold 0.0021 was required to keep the type I error rate at 5%. The p-values of the association analysis are presented in Table 2 as non-corrected. Out of 32 SNPs, four SNPs in *IL1R1 *gene in 125 kb area showed some evidence for association to hand OA (p-values < 0.05), in a family-based association analysis using the Pseudomarker program and/or in the case-control analysis (Table 2). Association with severe hand OA was concentrated in one LD block (r^2 ^> 0.34, D' > 0.94) harboring the area of long promoter and the coding region of gene *IL1R1 *(Fig. 2). It is notable, that association of the SNP rs2287047 to severe hand OA (odds ratio (OR) for genotypes AA vs. GG = 0.16, 95% confidence interval (CI) = 0.06 to 0.45; OR for genotypes AA vs. AG = 0.26, 95% CI = 0.09 to 0.73; protective A allele being the minor allele; p = 0.0009 dominant mode of inheritance) remained statistically significant after correction for multiple testing. This SNP is located in intron 1 of the *IL1R1 *gene. Association analysis using only female hand OA subjects provided comparable results (best p-value 0.0045 for SNP rs2287047, dominant model).

**Table 2:** Results of the single SNP association analysis of a 470 kb region on 2q11.2 in hand OA study sample.

		Controls	Hand OA		
			
				p-values	
				
Gene	SNP	MAF	MAF	χ^2^	Pseudomarker
**I**	rs740044	0.178	0.155	0.507^a^	0.517
	rs4141134	0.269	0.250	0.635^a^	0.327
***IL1R2***	rs719250	0.182	0.177	0.867	0.382
	rs3218934	0.406	0.456	0.246	0.086
	rs3218984	0.394	0.424	0.472	0.185
**II**	rs1008394	0.394	0.439	0.281	0.083
	rs2310173	0.304	0.351	0.226	0.370
***IL1R1***	rs1465325	0.195	0.119	**0.022^a^**	**0.005**
	rs956730	0.260	0.204	0.140^a^	**0.026**
	rs3917225	0.396	0.309	**0.043**	0.078
	rs2287047	0.249	0.159	**0.013^a^**	**0.0009^b^**
	rs3771200	0.411	0.453	0.312	0.539
***IL1RL2***	rs2241132	0.158	0.141	0.644^a^	0.491
	rs870684	0.378	0.394	0.690	0.781
	rs1922290	0.377	0.420	0.304	0.495
	rs1922295	0.379	0.400	0.613	0.696
	rs1997502	0.353	0.318	0.375	0.681
	rs2302612	0.119	0.157	0.199^a^	0.505
**III**	rs1558626	0.446	0.488	0.313	0.108
	rs1345302	0.447	0.414	0.428	0.205
	rs1882510	0.224	0.229	0.919^a^	0.517
	rs1420089	0.154	0.151	1.000^a^	1.000
***IL1RL1***	rs1997466	0.476	0.427	0.250	0.385
	rs1041973	0.202	0.173	0.458^a^	0.202
	rs12905	0.274	0.235	0.293	0.366
***IL18R1***	rs2287037	0.383	0.416	0.433	0.461
	rs2270298	0.265	0.189	0.081^a^	0.131
	rs1035130	0.277	0.241	0.338	0.453
	rs1420096	0.466	0.458	0.868	0.746
***IL18RAP***	rs1420106	0.192	0.200	0.832^a^	0.454
	rs1420100	0.469	0.447	0.599	1.000
	rs917997	0.189	0.185	1.000^a^	0.990

MAF = Minor allele frequency in the sample set

χ^2 ^= p-values from Pearson's χ^2 ^analysis of allele difference between cases and controls

Pseudomarker = p-values of the family-based association analysis with Pseudomarker (dominant mode of inheritance)

I, II and III = three intergenic regions

^a ^= calculated with Fisher's test

^b ^= p-value remained statistically significant after correcting for multiple testing

**Figure 3:**
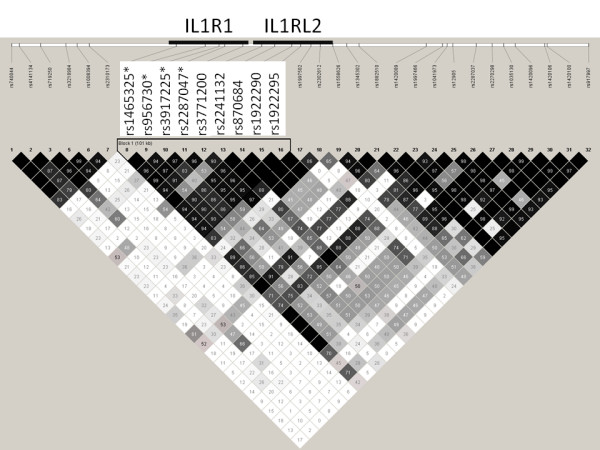
**LD structure and the degree of linkage disequilibrium (D') for each SNP pair in control sample **[30]. SNPs associating to hand OA (stars) and D' haplotype block.

One SNP (rs956730) in the same LD block showed nominal association in knee OA material (p = 0.045, minor allele frequency = 0.324) and further analysis using female knee OA subjects only provided some evidence for association with SNPs in the same LD block (r^2 ^= 0.17, D' = 0.90) (Fig. 3) mapping within the *IL1RL2 *gene (p-values 0.0098, 0.012 and 0.012 for SNPs rs870684, rs1922290 and rs1922295, respectively; dominant model). However, none of the associations were statistically significant after correcting for multiple testing.

At least 503 knee OA cases would have been required to have 90% power to detect association at the level of p < 0.001 for SNP rs2287047 when assuming a similar effect size as observed in the family-based severe hand OA (OR 22 vs. 11 = 0.16), whereas the power to detect association with the 113 knee OA cases was 12%. Most probably the effect size of the studied SNP (rs2287047) observed here is an over-estimate and if assuming an effect size typically seen in complex diseases (expected risk ratios (RRs) for a single SNP closer to 1.2 and 1.15, for AA and Aa, respectively), approximately 4000 cases would have been needed for detecting the association with 80% power (allele frequency of 0.75 and prevalence of 0.1) [32].

We were not able to detect association (Pseudomarker and Pearson's χ^2 ^tests) with any of the SNPs in the previously more intensively studied area (*TRAP1*, *IL1A*, *IL1B*, *IL1F5*, *IL1RN*) (p > 0.14). Also, opposite to findings by Smith et al.[29], we did not find LD between SNPs in *IL1R1 *and the locus containing *IL1A, IL1B*, and *IL1RN *in our study material (r^2 ^≤ 0.03, D'<0.54).

To cover most of the allelic variation on the genome region showing evidence for association to severe hand OA, we monitored for haplotype association for *IL1R1 *(rs1465325, rs956730, rs3917225, rs2287047, rs3771200) in hand and knee OA groups using the Haploview program [30] (Table 3). The frequencies of allele 3 (CAAAG) differed between hand OA cases (0.104) and controls (0.188) (p-value 0.008). SNP rs1465325 (OR CT vs. TT = 0.59, CI = 0.45 to 0.78; p = 0.022) tagged the protective allele. The protective A allele of SNP rs2287047 for hand OA was observed in two allelic backgrounds, on allelic haplotypes 3 and 5. Also, frequencies of the rare haplotype 5 (TAAAG) differed between knee OA cases (0.103) and controls (0.053) (p-value 0.004), but the haplotype 5 was the predisposing haplotype.

**Table 3:** IL1R1 haplotype frequencies in hand OA patients and in controls and the p-value for the difference (one allele versus all other alleles combined) based on the Haploview program [30].

		Allele frequenciesa		
		
**No**.	Allele	Controls	Hand OA^a^	p-value
1	TGGGA	0.381	0.440	0.147
2	TGGGG	0.218	0.212	0.865
3	CAAAG	**0.188**	**0.104**	**0.008**
4	TGAGG	0.115	0.123	0.772
5	TAAAG	0.053	0.048	0.796
6	others	-	-	

^a ^Only the index case of each hand OA family was included

Only haplotypes with frequency of over 0.05 in control sample are shown. The SNPs used to estimate the haplotypes were rs1465325, rs956730, rs3917225, rs2287047 and rs3771200, respectively.

Next, we estimated individual haplotypes with the Phase2 program [36,37], to calculate an odds ratio for the associated allelic haplotypes on basis of one allele versus all other alleles. We were able to estimate haplotypes fulfilling the preset criteria (probability ≥ 0.60) for 96.5% of the hand OA samples, 95.6% of the knee OA samples and 100% of the controls, and the frequencies of the estimated haplotypes were comparable to ones provided by Haploview. When comparing the hand OA material and controls, the associated protective allele 3 (CAAAG) provided an odds ratio of 0.54 (OR 3X vs. XX, 95% CI = 0.41 to 0.72) and the rare allele 5 (TAAAG) an odds ratio of 1.81 in the knee OA (OR 5X vs. XX, 95% CI = 1.34 to 2.43) where the X represents all other alleles combined. We were only able to calculate an odds ratio for one associated allele versus no associated alleles (3X vs. XX and 5X vs. XX) since there were no individuals homozygote for the associated allele either in the case groups or in the control group. The effect of allele 5 to knee OA was opposite to the effect for hand OA, but it is important to note that the allele 5 was rare.

We also performed a haplotype association test by selecting SNPs in D' LD with each other (measured by D'>0.94 for the first and the last SNP in the block; SNPs rs1465325, rs956730, rs3917225, rs2287047, rs3771200, rs2241132, rs870684, rs1922290 and rs1922295) in hand OA group and in controls. Allele CAAAGCTGC corresponding to allele 3 (CAAAG) in the previous test, showed similar allele frequencies, allele CAAAGCTGC being more rare in the hand OA cases (0.101) than in the controls (0.190) (p = 0.0058).

The four SNPs showing some association with hand OA provided also nominal evidence for linkage in the families (LOD = 0.640670, p = 0.042930 for rs1465325; LOD = 1.343799, p = 0.006440 for rs956730; LOD = 0.281669, p = 0.127359 for rs3917225; LOD = 0.841737, p = 0.024494 for rs2287047). Although all except one affected individual carried the predisposing common G-allele of rs2287047, GIST analysis resulted in non-significant p-values when using combined model of dominant, recessive and additive models (p = 0.672 for rs1465325, p = 0.499 for rs956730, p = 0.563 for rs3917225, p = 0.998 for rs2287047), suggesting that the SNPs in *IL1R1 *do not fully explain the originally observed linkage.

## Discussion

This study identifies an association between severe hand OA and the variants of the *IL1R1 *gene. The strongest evidence for association, which remained statistically significant after correction for multiple testing, was observed with SNP (rs2287047) located in the intron 1 of the *IL1R1 *gene. This gene represents a highly relevant biological candidate gene since it encodes protein that is a known modulator of inflammatory processes associated with joint destruction and resides within a locus providing consistent evidence for linkage to hand OA. *IL1R1 *did not show significant single SNP association with knee OA. It is possible that *IL1R1 *does not have strong or any affect in knee OA, or the lack of association for knee OA is due to lack of power in the analysis. Also the knee OA patients did not have a similar strong family background for the disease as the hand OA patients. However, nominal association in the same D' LD block in *IL1RL2 *gene was observed for knee OA in females. Further, the association between radiological bilateral DIP OA (K/L at least 2) and *IL1R1 *and *IL1RL2 *genes was recently studied also by Solovieva et al. [42] in study material of 206 cases and 328 controls. None of the single SNPs (rs1465325, rs956730, rs2287047) showed association to bilateral DIP OA, but some occupation dependent association was observed with a haplotype covering the *IL1R1 *and *IL1RL2 *genes. However, it is worth of noting that the phenotype utilized in the study by Solovieva and collaborators is not equivalent to the phenotype used here (severe bilateral hand OA) and that approximately 4000 cases would be required to exclude SNPs with the effect size typically observed in complex diseases.

There are some limitations in the present study. Based on the GIST analysis, the observed associations for single SNPs in this study does not fully explain the linkage obtained in the previous study using microsatellite markers. It is likely that the SNPs associated with hand OA in this study are not the causative variants but are probably in LD with the true causative variant(s) located in the *IL1R1 *gene region. Furthermore, it is plausible that allelic variants in several genes within this locus contribute to OA susceptibility, each with a small effect. Other studies seem to support this hypothesis: The interleukin 1 gene family has recently been a widely studied target in osteoarthritis [8,10-12,29,43-46], partly due to several genome wide scans demonstrating linkage to this region [6-8], but also because of the potential role of the gene products in inflammatory responses associated with the development of OA [13,15,16,47,48]. Mostly, the previous association analyses have concentrated on the genes *IL1A*, *IL1B *and *IL1RN *and have left the 470 kb region targeted in this study, with less attention. However, we did not see association in the previously more intensively studied area harbouring the *IL1A*, *IL1B *and *IL1RN *genes using the Finnish hand and knee OA materials.

The limited number of subjects with severe hand OA (Table 1) is another limitation in this study. However, it is important to emphasize the unique nature of this study sample as its strength: the hand OA study sample is based on the same material, which provided evidence for linkage to this genome region on 2q in our genome wide scan. Additionally, an association analysis with the Pseudomarker program, which can utilize information from several family members in addition to case-control data, provided the strongest evidence for association. Using the family based analysis putatively gives us the advantage to identify population specific variants that are important in understanding the aetiology of the disease. Further, most subjects in the hand OA material have a severe end stage disease and very strict criteria was used in selecting the study sample. Sequencing of the associated region in these familial hand OA cases may identify rare, potentially causative variant(s) of the gene segregating with the common allelic variant in these families. Thus, further validation in a large sample set and additional biological evidence are required to confirm the role of *IL1R1 *in severe hand OA.

A complex formed by IL-1, IL1R1 and IL1RAP initiates numerous signalling pathways (see review [13]). *IL1R1 *has three alternative promoters generating alternative transcripts [49]. The promoter region of *IL1R1 *has proven to be highly polymorphic, possibly due to the polymorphisms affecting the degree of expression in a wide variety of tissues [29]. IL1R1 is not abundant in cells but less than 10 ligand-occupied receptor molecules on the cell's surface is enough to induce a strong response due to parallel signalling pathways [50]. One of these pathways leads to activation of NF-κB, a regulator of inflammatory and immune gene expression [15]. Lately, the contribution of NF-κB signalling components to the pathogenesis of different rheumatic diseases and the pharmacologic modulation of NF-κB has been widely studied. These studies have yielded promising results such as currently available therapeutic agents, novel small molecule inhibitors and improved antisense DNA therapy and RNA interference [51]. It has been shown, that blocking the signal transduction of IL1R1 prevents OA progression in animal models [52]. This would support the biological significance of our finding of *IL1R1 *as a potential factor in OA development. All four associated SNPs were located in the non-coding region of *IL1R1*, but at least three of them (rs1465325, rs3917225 and rs2287047) co-localized with predicted transcription factor binding sites so that the conserved allele matched with the consensus sequence of the transcription factor binding site while the other allele did not or changed it to other transcription factor binding site (Computational Biology Research Center CBRC, http://www.cbrc.jp/index.eng.html). Polymorphisms in *IL1R1 *gene has been associated with other diseases as well, for instance AIDS progression [53], endometriosis [54] and Helicobacter pylori infection [55].

## Conclusions

To summarize, a set of 32 SNPs mapping within a 470 kb region surrounding the strongest linkage peak of a genome wide scan performed in Finnish severe hand OA families was genotyped in 134 hand and 113 knee OA cases and 436 controls. The family based association analysis demonstrated association to four SNPs mapping to a 125 kb DNA region comprising the *IL1R1 *gene. The strongest evidence for association, observed with a SNP rs2287047 located in the intron 1 of the *IL1RL1 *gene (p = 0.0009) remained significant after correction for multiple testing. The haplotype analysis provided further evidence for the *IL1R1 *gene: One haplotype allele showed association with hand OA. The *IL1R1 *gene encodes receptor whose activation by the binding of a specific ligand leads to activation of NF-κB, which is a modulator of inflammatory and immune gene expression. However, the variants linked and associated with hand OA in this study did not fully explain the original linkage observed in our previous study suggesting that there may also be other hand OA predisposing variants within this locus. Potentially chromosome region 2q harbors several variants in different genes affecting the disease.

## Competing interests

The authors declare that they have no competing interests.

## Authors' contributions

JS, JK, LP and UMK designed and coordinated the study and participated in writing the manuscript. SK carried out the genotyping. AN and SK participated in designing the study, carried out all the statistical analyses and drafted the manuscript. AH, KT and UMK are responsible for the phenotyping and collection of the samples, and they participated in writing the manuscript. TV, MCB and JK are responsible for the control samples and participated in writing the manuscript. All authors read and approved the final manuscript.

## Pre-publication history

The pre-publication history for this paper can be accessed here:

http://www.biomedcentral.com/1471-2350/11/50/prepub
